# TIMP-1 Inhibits Apoptosis in Lung Adenocarcinoma Cells via Interaction with Bcl-2

**DOI:** 10.1371/journal.pone.0137673

**Published:** 2015-09-14

**Authors:** Srilatha Nalluri, Sampa Ghoshal-Gupta, Ammar Kutiyanawalla, Sitaram Gayatri, Byung Rho Lee, Shahanawaz Jiwani, Amyn M. Rojiani, Mumtaz V. Rojiani

**Affiliations:** Department of Pathology, Georgia Regents University-Medical College of Georgia, Augusta, GA, United States of America; Wayne State University School of Medicine, UNITED STATES

## Abstract

Tissue inhibitors of metalloproteinases (TIMPs) are multifaceted molecules that exhibit properties beyond their classical proteinase inhibitory function. Although TIMP-1 is a known inhibitor of apoptosis in mammalian cells, the mechanisms by which it exerts its effects are not well-established. Our earlier studies using H2009 lung adenocarcinoma cells, implanted in the CNS, showed that TIMP-1 overexpressing H2009 cells (HB-1), resulted in more aggressive tumor kinetics and increased vasculature. The present study was undertaken to elucidate the role of TIMP-1 in the context of apoptosis, using the same lung cancer cell lines. Overexpressing TIMP-1 in a lung adenocarcinoma cell line H2009 resulted in an approximately 3-fold increased expression of Bcl-2, with a marked reduction in apoptosis upon staurosporine treatment. This was an MMP-independent function as a clone expressing TIMP-1 mutant T2G, lacking MMP inhibition activity, inhibited apoptosis as strongly as TIMP1 overexpressing clones, as determined by inhibition of PARP cleavage. Immunoprecipitation of Bcl-2 from cell lysates also co-immunoprecipitated TIMP-1, indicative of an interaction between these two proteins. This interaction was specific for TIMP-1 as TIMP-2 was not present in the Bcl-2 pull-down. Additionally, we show a co-dependency of TIMP-1 and Bcl-2 RNA and protein levels, such that abrogating Bcl-2 causes a downregulation of TIMP-1 but not TIMP-2. Finally, we demonstrate that TIMP-1 dependent inhibition of apoptosis occurs through p90RSK, with phosphorylation of the pro-apoptotic protein BAD at serine 112, ultimately reducing Bax levels and increasing mitochondrial permeability. Together, these studies define TIMP-1 as an important cancer biomarker and demonstrate the potential TIMP-1 as a crucial therapeutic target.

## Introduction

The American Cancer Society 2014 report estimates 224,210 new lung and bronchial cancer cases in the USA alone with an estimated 159,260 deaths [1]. Lung cancer is classified as small cell (approximately 15%) and non-small cell (approximately 85%) and is the leading cause of cancer related mortality [2]. An important mechanism in the process of cancer progression and metastasis of tumor cells involves the degradation of tissue barriers in the extracellular matrix, particularly the basement membrane by matrix metalloproteinases (MMPs). These proteases are kept in check by their endogenous physiological inhibitors i.e. tissue inhibitors of metalloproteinases (TIMPs). Over the years, the 4 different isoforms of TIMPs have been shown to be multifunctional proteins affecting tumor growth, apoptosis and angiogenesis. TIMP-3 induces apoptosis and inhibits angiogenesis [3], whereas TIMP-2 and TIMP-4 have been shown to be both, pro and anti-apoptotic [4–7]. TIMP-1 the most-studied of all the members, was originally identified with erythroid-potentiating activity and has since been documented to be growth-promoting for a number of cell lines [8]. It has also been shown to be either a positive or a negative regulator of angiogenesis [9–11].

In clinical studies, high serum levels of TIMP-1 in patients with a variety of cancers have been associated with poor prognosis. This has been demonstrated through substantial data on breast cancer [12], gastric cancer [13] and colorectal cancer [14]. TIMP-1 has also been shown to be of prognostic value in NSCLC [15, 16]. Recently, it has been inferred that TIMP-1 may also have predictive value in defining response to chemotherapeutic agents [17].

Numerous studies have documented the well-established anti-apoptotic function of TIMP-1[18]. TIMP-1 has been shown to protect breast epithelial cells against intrinsic and extrinsic cell death involving FAK/PI3 kinase and ERK [19, 20]. Overexpression of the anti-apoptotic molecule Bcl-2 has also been documented to increase TIMP-1 expression [21]. In Burkitt’s lymphoma cell lines, TIMP-1 expression suppressed apoptosis and upregulated Bcl-xL [22]. In mouse bone marrow stromal cell line, recombinant TIMP-1 inhibited apoptosis by increasing the expression of Bcl-2 and decreasing Bax expression [23]. These studies have documented the antiapoptotic function of TIMP-1 to be independent of its MMP inhibitory activity, although MMP-dependent functions have also been shown [18, 24].

In the present study, we have investigated the role of TIMP-1 overexpression in H2009, lung adenocarcinoma cell line. We present evidence that TIMP-1 overexpression increases levels of Bcl-2 resulting in inhibition of apoptosis via inactivation of BAD following its phosphorylation at serine 112. This inhibition of apoptosis occurs through the p90RSK/BAD axis via an interaction between TIMP-1 and Bcl-2. We further show evidence of the existence of a coordinated loop controlling the levels of TIMP-1 and Bcl-2 interdependently.

## Methods

### Cell Lines and Cell Culture

NCI-H2009 cells were maintained in RPMI 1640 medium supplemented with 10% fetal bovine serum and 100 μg/ml gentamycin and the TIMP-1 overexpressing H2009 clones as well as empty vector clones received 6μg/ml G418. Generation of stable TIMP-1 overexpressing H2009 clones HB-1 and HB-6 were as described previously [25]. All cells were cultured in the presence of 5% CO_2_ and 95% humidity at 37°C.

### Reagents and Antibodies

Staurosporine (Cell signaling, Danvers, MA) and ABT 737 (Chemie Tek, Indianapolis, IN) were dissolved in DMSO to produce 10 mM and 100 mM stock solution respectively; complete mini protease inhibitors and Phos stop (Roche, Mannheim, Germany) were prepared according to manufacturer’s protocol.

The primary antibodies used are Anti-TIMP-1 (Millipore, California, USA), anti-Bcl-2, Bax, BAD, PARP, p90RSK (Cell Signaling, Danvers, MA) and anti-Actin from SIGMA (St. Louis, MO). The primary antibodies were used at 1:1000 dilutions unless mentioned otherwise. The Quantikine ELISA kit (R&D system, Minneapolis, MN) was used to confirm the amount of endogenous and secreted TIMP-1 in the parent cell line and the clones.

### Cell Survival Assay

To examine the effect of TIMP-1 on apoptosis sub-confluent cells were treated with 1μM ABT-737 and 0.5 μM Staurosporine. Cell viability was determined by Trypan Blue assay (25).

### Hoechst 33258 Assay

Cells were plated in 24-well plates and incubated for 24 h. Treatments were then added to each well according to the experimental groups, and incubated for 24 h. The cells were washed with PBS three times and stained with Hoechst 33258 (1 mg/l) for 10 min at 37°C. The cells were again washed with PBS three times and images of the Hoechst 33258 fluorescence were captured using an inverted fluorescence microscope. Apoptosis was calculated as follows: 400 cells were randomly counted with an optical microscope at 200× magnification and the apoptotic rate was expressed as apoptotic cell number/total cell number ×100%. A mean value was obtained from three parallel wells.

### Apoptosis Specific Gene Array

PCR gene array specific for apoptosis was purchased from SA Biosciences (Catalog # PAHS-012). The protocol was followed as per the manufacturer’s instructions.

### ELISA

Changes in extracellular and intracellular concentrations of TIMP-1 were determined using a commercially available ELISA kit (R&D systems, Minneapolis, MN). Briefly, TIMP-1 in cell culture media and in lysates were measured according to manufacturer’s instructions. Each individual assay was performed in triplicate.

### Immunoblot Analysis

Cells were seeded in tissue culture dishes at a density of 3x10^4^ cells /cm^2^. Protein extracts were prepared at the indicated time points using RIPA lysis buffer containing complete mini, phos-stop (Roche) and PMSF (1 mM, SIGMA). The protein concentration in each sample was determined by BCA Protein Assay kit (Pierce, Rockford, IL, USA). Proteins were separated by sodium dodecyl sulphate (SDS)–gel electrophoresis using 10–12% polyacrylamide gels and blotted on to PVDF membranes (Bio-RAD). The membranes were blocked in washing buffer (Tris-buffered saline (PBS) + 0.1% Tween 20) containing 5% dry milk or 5% Bovine serum albumin and incubated with the primary antibody. Subsequently, the blots were washed 3 × 10 min in washing buffer followed by incubation with the appropriate horseradish peroxidase-conjugated secondary antibody. Following 3 × 10 min washes in washing buffer, the blots were developed by the chemiluminescent detection system (Denville Scientific, NJ) according to the manufacturer's instructions. In order to obtain a loading control, the blots were stripped and re-probed with a primary monoclonal antibody recognizing β-actin (SIGMA), diluted 1: 10,000 in washing buffer containing 1% dry milk. Finally, the blots were washed 3 × 10 min in washing buffer and developed as described above. Serum-free conditioned media (SFCM) were collected for each clone, concentrated and protein estimation done by BCA assay (Pierce). For SFCM, 20 μg of protein was loaded and subjected to SDS-PAGE under non-reduced conditions and transferred to PVDF membrane and immunoblot analysis was done as above.

### Quantitative Real-Time RT-PCR

Total RNA was isolated from the cells using RNeasy mini kit from Qiagen (Maryland, USA). Equal amounts of RNA were used to generate the first strand cDNA (iscript Reverse Transcription Supermix, Biorad), and quantitative real-time PCR was performed on the Biorad CFX connect PCR system using Sybr Green qPCR Master Mix (Biorad, USA). The relative expression levels of target genes were analyzed by examining the mRNA expression of each target gene normalized to GAPDH. Error bars represent Standard Error of Mean (SEM) of three independent experiments.

### Immunoprecipitation

Total cell lysates were prepared as described under Western blot procedure. To preclear the lysate, approximately 1 ml (1000μg) of whole cell lysate was mixed with 0.25 μg of the appropriate control IgG (corresponding to the host species of the primary antibody) together with 20 μl of protein A/G agarose (25% v/v), and incubated at 4°C with rotation for 30 min. Primary antibody (10μg) was added to the precleared lysates and incubated at 4°C for 4 hours. 20 μl of agarose beads were added to the mixture and incubated at 4°C overnight on a rotating device. The pellets were collected by centrifugation (1000xg at 4°C) and washed 3 times with RIPA buffer. The final wash was in PBS and the pellet was resuspended in Laemlli sample buffer. The samples were boiled for 3 min and subjected to electrophoresis and immunoblot analysis.

### T2G Mutants

The TIMP-1cDNA was constructed by the polymerase chain reaction using primers TIMP-1, B*am*HI-ATG, 5′- tgtatggatccaccATGGCCCCCTTTGAGCCCCTGG-3′, and TIMP-1-HindIII-R, 5′- ctacgaagcttTCAGGCTATCTGGGACCGCAGGGAC-3’n from the full-length TIMP-1 cDNA fragment from pBK-CMV vector used previously in our lab [25]. To substitute Thr-2 with Gly at the mature TIMP-1 protein, site-directed mutagenesis was performed using primers TIMP-1-T2G-mut-F, CCAGCAGGGCCTGCggCTGTGTCCCACCCCAC and TIMP-1-T2G-mut-R, GTGGGGTGGGACACAGccGCAGGCCCTGCTGG. DNA sequencing analysis confirmed the fidelity of the constructs. Hereafter, the mutated TIMP-1 constructs are referred to as T2G TIMP-1.

### Statistical Analysis

All experiments were repeated 3–5 times. Significant differences between sets of values for control and test groups were assessed by appropriate statistical analysis. Statistical significance was determined by ANOVA for multiple comparisons and student's t-test for two groups using Graphpad Prism 6 software. A p-value significance was set at P<0.05 to compare the measured parameter of an experimental group with that of its control.

## Results

### 1. Overexpression of TIMP-1 in NCI-H2009 Cells Results in Up-Regulation of Bcl-2 and Protects against Staurosporine Induced Apoptosis

**Fig 1:**
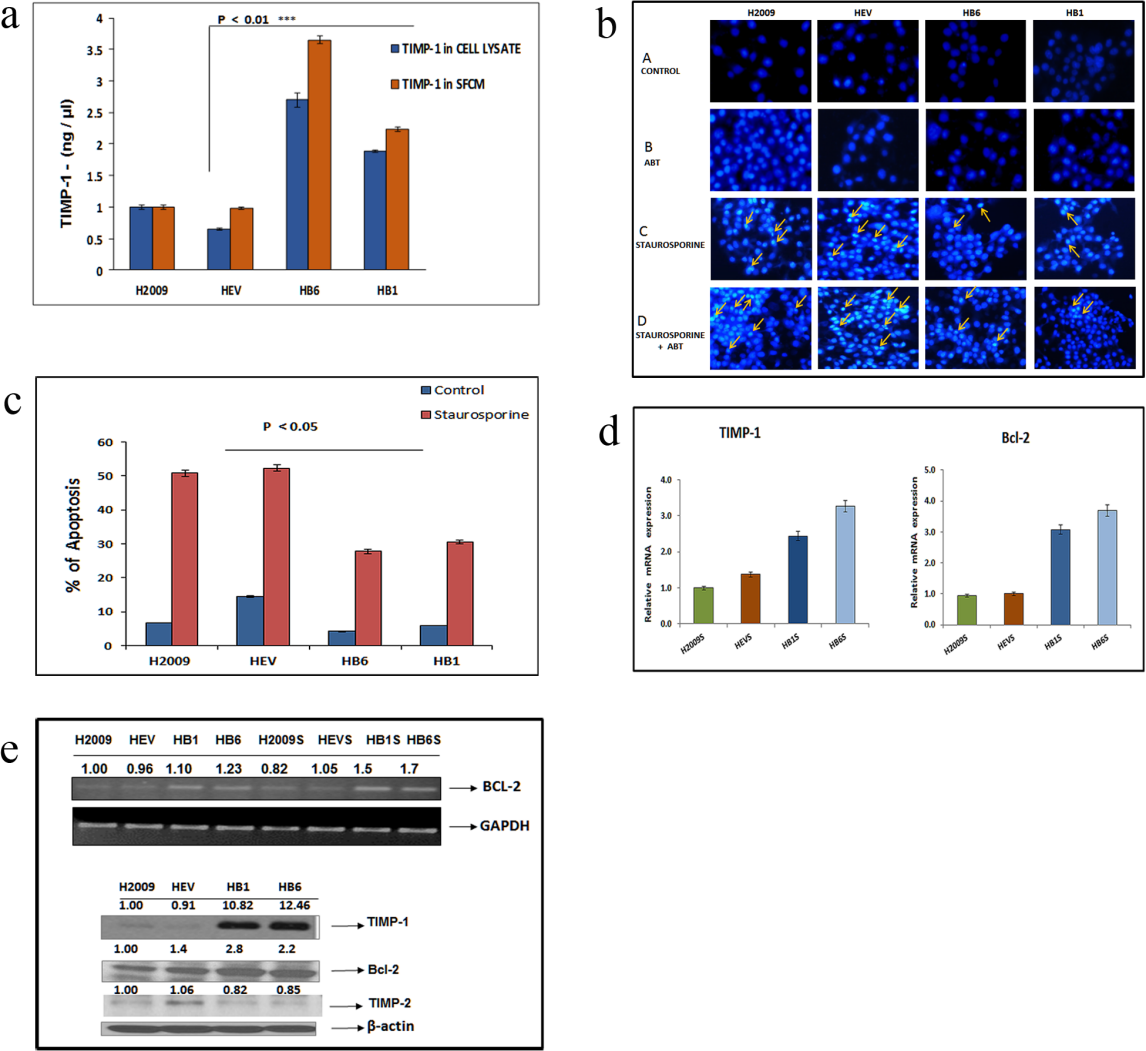
(a) Comparison of exogenous (in SFCM) and endogenous (cell lysates) TIMP-1 in H2009, empty vector and TIMP-1 overexpressing clones: Cells were grown overnight in serum free media to assess exogenous TIMP-1 and cell lysates were collected from cells grown in complete media. The conditioned media and the cell lysates from the cell lines were assayed for TIMP-1 by ELISA. The X-axis shows H2009 cell line and its clones. The Y-axis shows concentration of TIMP-1 in ng/mL. A significant upregulation of TIMP-1 in overexpressed clones is seen (p<0.01, Student’s t test). Data represents independent triplicate determinations ± SEM (standard error of mean). (b) Effect of ABT-737 and Staurosporine on apoptosis: Cells were first treated with ABT-737 (1 μM, for 21 hours) following which Staurosporine was added at 0.5μM concentration for 3 hours (all apoptosis related experiments follow this regimen unless mentioned otherwise). Cell apoptosis was evaluated by Hoechst 33258 staining. The control cells displayed normal nuclei. Staurosporine treatment with or without ABT-737 showed significantly more condensed and bright fluorescent nuclei with nuclear fragmentation in H2009 and HEV cells. The number of apoptotic cells were less in the HB1 and HB6 cells in panel C and D as shown by the arrows. (c) Quantitative representation of Hoechst 33258 apoptosis assay: Hoechst staining showed significantly more apoptosis in H2009 cells and the empty vector clones (HEV), compared to the TIMP-1 overexpressing clones(HB1, HB6), by One Way ANOVA (p<0.05), with or without staurosporine treatment. The control groups (H2009, HEV) showed almost 2-fold greater apoptotic morphology compared to TIMP-1 overexpressing clones. Data is representative of 3 independent experiments ± SEM. (d) TIMP-1 overexpressing cell lines show increased expression of Bcl-2 mRNA in response to staurosporine treatment: H2009 cells and its clones were treated with 0.5μM Staurosporine (S) and subjected to apoptosis specific PCR array. A significant 3 fold increase in Bcl-2 and TIMP1, was observed and confirmed by qPCR (p<0.01, One Way ANOVA with posthoc Dunnett’s test). (e) Expression of Bcl-2 and TIMP-1 in H2009 cells: The top panel shows semiquantitative reverse transcription PCR after 3hrs of Staurosporine (S) treatment. An increased amount of Bcl-2 is observed in the TIMP-1 overexpressing clones. Lower panel indicates an increased amount of Bcl-2 in the TIMP-1 overexpressing clones by western blot. The TIMP-2 level remained unchanged. The relative protein band density was normalized to β-Actin. Data is representative of triplicate independent experiments.

Previously, we have shown that overexpressing TIMP-1 by stably transfecting pBK-CMV-TIMP-1 plasmid in lung adenocarcinoma cell line NCI-H2009 injected into the mouse brain, resulted in aggressive tumors with increased microvessel density [25]. To investigate the role of TIMP-1 in cell survival we first confirmed the TIMP-1 expression levels in H2009 cells and its overexpressed clones (described in materials and methods) by enzyme linked immunosorbent assay (ELISA) as represented in Fig 1A. Endogenous TIMP-1 levels in HB1 and HB6 were ~2–3 folds higher, while the secretory TIMP-1 levels in the overexpressed clones were ~2–4 fold more compared to the controls. Expression of TIMP-1 was significantly increased in the overexpressed clones vs. empty vector clones (p value <0.01, Student’s t test). As TIMP-1 is a secreted protein, TIMP-1 secreted levels are always higher compared to endogenous levels as seen.

H2009 cells and its clones were treated with Staurosporine (0.5 μM) and subjected to apoptosis specific PCR array to identify induction of apoptosis specific genes. A three-fold increase in Bcl-2 was observed in the TIMP-1 overexpressing clones, which was also confirmed by PCR assays.

Since TIMP-1 overexpression enhanced Bcl-2 expression, we examined the effect of Staurosporine induced apoptosis in H2009 cells and its TIMP-1 overexpressing clones by quantifying the number of pyknotic or condensed nuclei visualized by Hoechst 33258 staining. Staurosporine was used at a concentration of 0.5 μM for 3 hours, to induce apoptosis in these cells without any cell death (data not shown).

Hoechst 33258 staining was used to visualize nuclear changes such as fragmentation and condensation that are characteristic of apoptosis (Fig 1B). The cells overexpressing TIMP-1 (HB1 and HB6) appeared less condensed compared to parental and empty vector clones, though abrogation of Bcl-2 by the BH-3 mimetic ABT-737 rendered the HB1 and HB6 clones more apoptotic with 0.5 μM Staurosporine treatment for 3 hours. Quantitative analysis showed considerably more cell death and condensed nuclei in H2009 and the empty vector control compared to the TIMP-1 overexpressing clones. (p = 0.01) Fig 1C. These findings demonstrate that TIMP-1 protects against apoptosis. Next, mRNA levels in the cells were confirmed by end-point PCR (Fig 1D), and Western blot analysis also revealed an elevation in the Bcl-2 protein expression level in the TIMP-1 overexpressing clones (Fig 1E).

### 2. TIMP-1 Affects PARP Cleavage and Caspase-3 Activity via Bcl-2 Activation; Inhibiting Bcl-2 Restores PARP Cleavage in TIMP-1 Over-Expressing Clones

**Fig 2:**
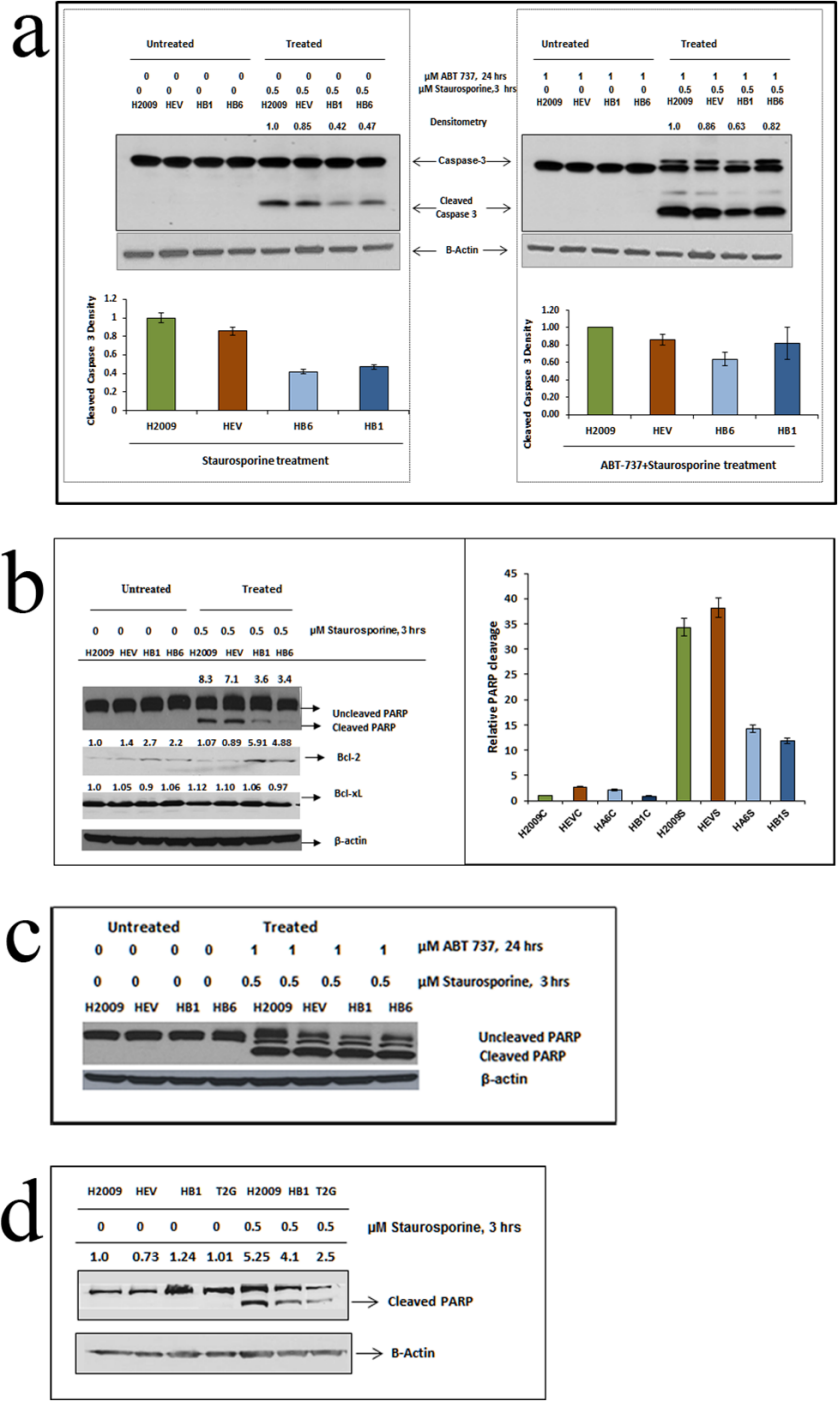
(a) Caspase-3 cleavage after Staurosporine treatment: The left panel shows Caspase-3 cleavage in H2009 and its clones treated with 0.5μM Staurosporine for 3 hours. The right panel shows Caspase-3 cleavage after ABT-737 and Staurosporine treatment, where more caspase cleavage is observed in HB6 and HB1. Graph represents densitometric analysis of the cleaved caspase western blot in each panel. Data represents independent triplicate determinations ± SEM (standard error of mean). (b) TIMP-1 inhibits apoptosis induced by Staurosporine: TIMP-1 overexpression does not result in significant PARP-1 cleavage after 3 hrs of 0.5μM Staurosporine treatment, probably due to upregulation of Bcl-2, which plays an inhibitory role. Expression of Bcl-xL was unchanged. The side panel shows the graphical representation of cleaved PARP expression (p<0.0001, ANOVA). Data is representative of three independent experiments. C = control, S = Staurosporine treated. (c) Abrogating Bcl-2 by ABT-737 restores the PARP cleavage pattern: Treatment with 1μM ABT-737 for 24 hrs followed by 0.5μM Staurosporine from 21^st^ hour increased PARP cleavage in HB1 and HB6 cells. (d) Antiapoptotic function of TIMP-1 is independent of MMP activity: The T2G mutated TIMP-1 overexpressing clone showed similar pattern of PARP cleavage indicating that this activity of TIMP-1 was MMP independent.

One of the key events executing the apoptosis commitment step is the activation of zymogen caspases following release of cytochrome c from the mitochondria, during intrinsic apoptosis. Staurosporine is known to induce cell death either through intrinsic or non-apoptotic pathways [26]. To confirm Bcl-2 associated apoptosis, we analysed the expression of caspase-3 as well as its substrate PARP-1. Apoptosis induced by Staurosporine followed by western blot analysis showed a marked reduction in Caspase-3 cleavage as well as PARP-1 cleavage in TIMP-1 overexpressing clones compared to control (Fig 2A and 2B). Inhibition of Bcl-2 activity by ABT-737 (1μM) for 24 hours followed by treatment with Staurosporine resulted in restoration of Caspase-3 cleavage as shown in Fig 2A, bottom panel. Similarly, reduced PARP-1 cleavage in TIMP-1 overexpressing clones was restored following ABT-737 treatment (Fig 2C). Bcl-2 is a better target of ABT-737 than Bcl-xL and Bcl-w [27, 28], hence our results indicate that upregulation of TIMP-1 is playing a role in apoptosis in a Bcl-2-dependent manner.

Furthermore, to elucidate if the antiapoptotic function seen in our system was the result of TIMP-1 interactions and was not MMP-mediated, as has been previously reported [19, 29], we generated a mutant form of TIMP-1. Altering the second amino acid threonine to glycine (T2G), results in impaired MMP-inhibitory function. The T2G mutants, like TIMP-1 overexpressing clones, inhibited apoptosis as seen by reduced PARP cleavage (Fig 2D).

### 3. TIMP-1 Interacts with Bcl-2 in Exerting Its Anti-Apoptotic Role and Contributes to a Feedback Loop in H2009 Lung Cancer Cells

**Fig 3:**
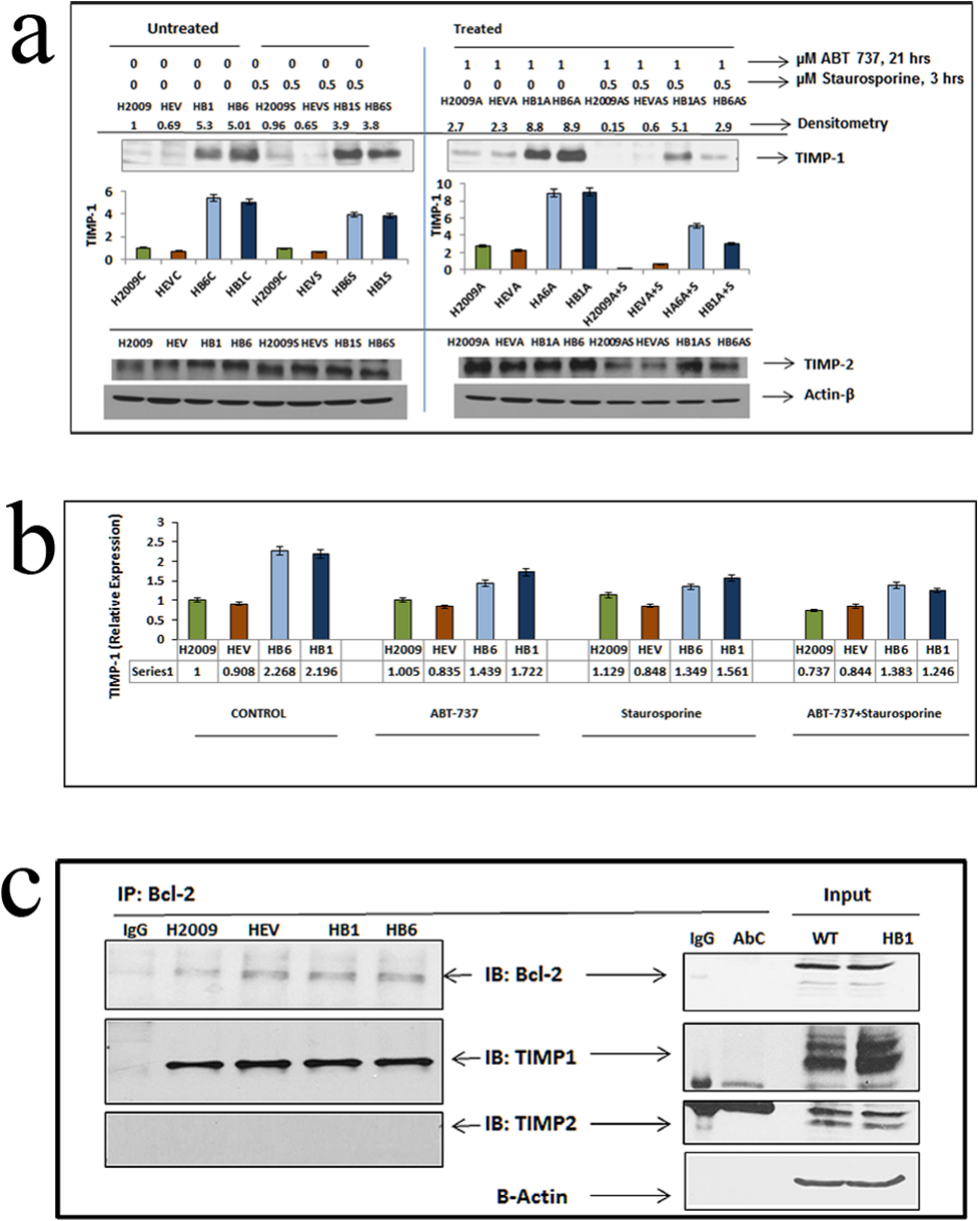
(a): Interdependency of TIMP-1 and Bcl-2 proteins: H2009 cells and its clones were treated with 0.5μM Staurosporine (S), which showed a reduction of TIMP-1 protein expression. Treatment with a BH3 mimetic ABT-737(A) reduced TIMP-1 protein levels compared to the controls. Combined treatment with ABT-737 and Staurosporine (AS) showed a marked reduction of TIMP-1 expression, without any changes in TIMP-2. (b) TIMP-1 mRNA expression level after treatment with Staurosporine and ABT-737: HB1 and HB6 TIMP-1 levels were reduced with combined ABT-737 and Staurosporine treatment. Two Way Repeated Measure ANOVA showed p = 0.0063, indicating a very significant down regulation of relative expression of TIMP1 in the overexpressed clones after ABT+Staurosporine treatment indicating down-regulating Bcl2 affects TIMP1 level. (c) Co-immunoprecipitation of TIMP-1 and Bcl-2: A possible interaction between the Bcl-2 and TIMP-1 revealed by co-immunoprecipitation. Cell extracts from H2009 and it’s clones were immunoprecipitated (IP) with anti-Bcl-2 or normal rabbit IgG as a negative control. The immunoblot (IB) was probed for TIMP-1, TIMP-2 and Bcl-2. No TIMP-2 was pulled down with Bcl-2, showing that only TIMP-1 is associated with Bcl-2 when apoptosis is induced. Input represents 60μg of cell extract.

Several studies have shown that either overexpressing Bcl-2 results in TIMP-1 up-regulation [21, 30] or that TIMP-1 treatment of cells results in increased Bcl-2 levels [31, 32]. Since overexpression of TIMP-1 up-regulated Bcl-2, and abrogating Bcl-2 enhanced apoptosis, we hypothesized abrogation of Bcl-2 may affect TIMP-1 levels. We therefore treated the cells with 1μM ABT-737 for 21 hours and 0.5μM Staurosporine for the last 3 hours and assessed mRNA and protein expression levels at 24 hour time point. Upon inhibition of Bcl-2 with ABT-737 during apoptosis induction, TIMP-1 protein and RNA levels were reduced (Fig 3A and 3B). These results exhibit an interdependent relationship between Bcl-2 and TIMP-1 in these cells. This interaction was specific for TIMP-1 as TIMP-2 levels were not concurrently altered (Fig 3A). This feedback loop between TIMP-1 and Bcl-2 led us to hypothesize that TIMP-1 may directly or indirectly interact with Bcl-2 to exert its anti-apoptotic effect.

Our studies revealed that immunoprecipitation of Bcl-2 from cell lysates also co-immunoprecipitated TIMP-1, thereby reinforcing this notion of an interaction between Bcl-2 and TIMP-1. This co-immunoprecipitation was specific to TIMP-1 as TIMP-2 was absent from the Bcl-2 pull-down (Fig 3C).

### 4. Overexpression of TIMP-1 Inhibits Apoptosis through Up-Regulated Bcl-2 and Acts via the p90RSK/pBAD-Ser112 and Not through AKT/pBAD-Ser136 Axis

**Fig 4:**
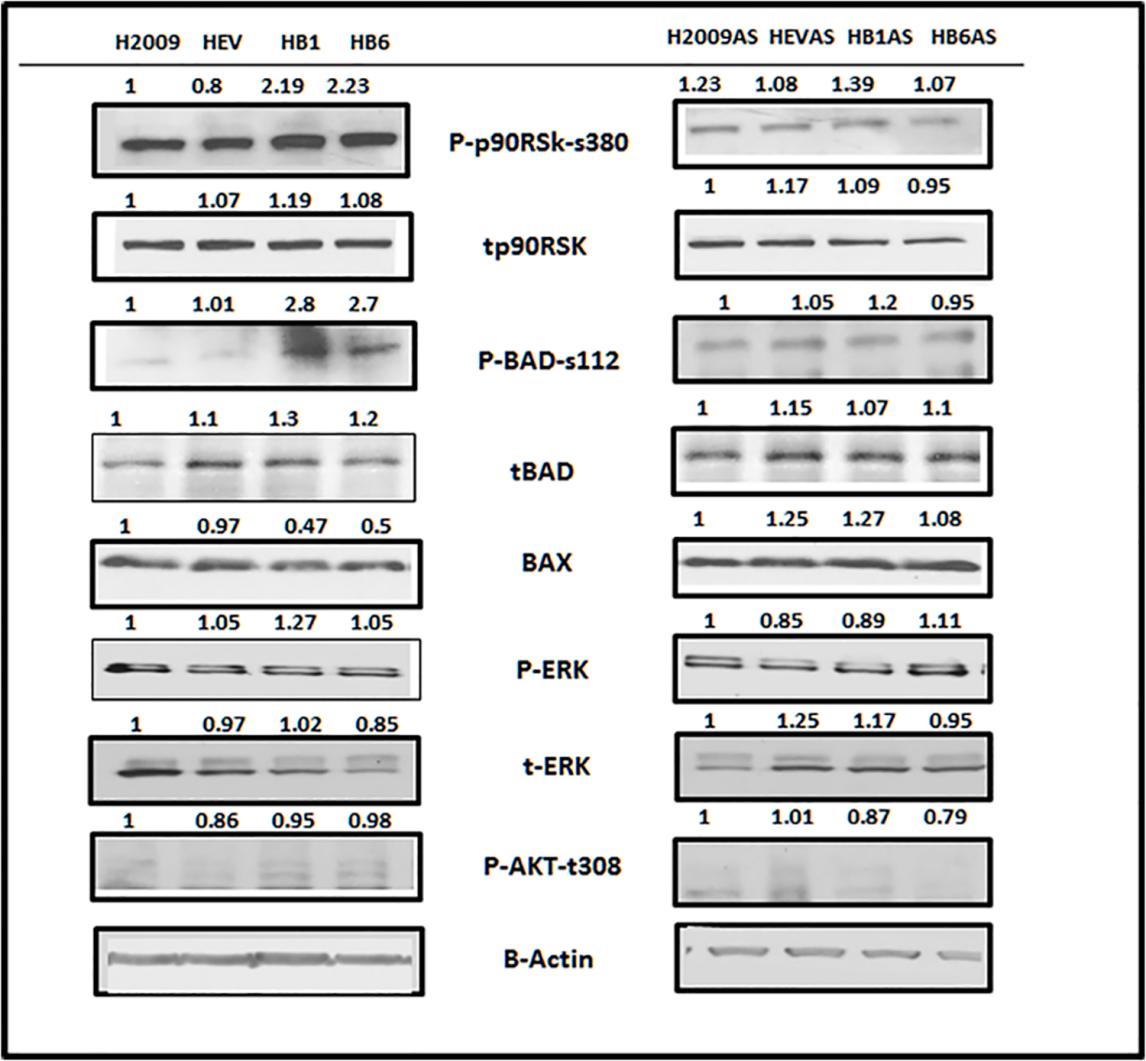
Antiapoptotic function of TIMP-1 does not involve the Akt pathway. TIMP-1 overexpression activates p90RSK which in turn activates BAD, concomitantly reducing Bax expression in the H2009 cells. The right panel shows treatment with ABT-737 and Staurosporine (AS) restores the p-p90RSK, p-BAD and Bax levels. Fig 4 also shows absence of Akt involvement.

The decision to undergo apoptosis is ultimately determined by the balance between pro-apoptotic and anti-apoptotic proteins. To determine the molecular mechanism underlying resistance to staurosporine induced apoptosis in our cells, we next tried to identify the levels of pro death or pro survival proteins that were involved in inhibiting the intrinsic apoptotic pathway. BAD, a pro-apoptotic member, forms heterodimers with Bcl-2 or Bcl-xL thereby incapacitating their anti-apoptotic functions. In the TIMP-1 overexpressing H2009 clones, BAD phosphorylation at Ser-112 was evident with no Ser-136 phosphorylation (Fig 4, left). Since phosphorylation of BAD at Ser-136 is mediated through Akt, we assessed Akt phosphorylation at both Ser -473 and Thr-308, which did not show any activation (Fig 4) indicating that the Akt pathway was not affected by TIMP-1 and that BAD was being phosphorylated by a molecule other than Akt. In fact overexpression of p90RSK, a serine threonine kinase, is known to phosphorylate BAD at Ser-112. As shown in Fig 4 p90RSK is being phosphorylated at Ser-380 resulting in its activation in the TIMP-1 overexpressing clones. Additionally the level of pro-apoptotic protein Bax was down regulated in these HB1 and HB6 clones, further indicative of resistance to apoptosis in H2009 clones (Fig 4).

## Discussion

This study was undertaken to define the role of TIMP-1 in lung adenocarcinoma cell line. Although TIMP-1 is emerging as an important prognostic marker in clinical findings and several studies have documented the prognostic value of TIMP-1 in lung cancers [16, 33, 34], the antiapoptotic role of TIMP-1 has not been robustly addressed in lung adenocarcinoma cell lines, with the majority of studies having been carried out using the A549 cell line with conflicting results [35, 36].

Earlier studies, documenting the antiapoptotic function of TIMP-1 have correlated higher TIMP-1 levels with either increased expression of Bcl-xL [22, 37, 38] or to that of Bcl-2 [21, 23, 32] or both [29]. Liu et al [20] have shown that TIMP-1 inhibits apoptosis by inducing specific cell survival pathways. It has also been shown that Bcl-2 overexpression results in increased TIMP-1 expression in breast epithelial cell lines, consequently suggesting that the antiapoptotic activity of TIMP-1 is independent of its ability to stabilize cell-matrix interactions [21]. In our study we found increased expression of Bcl-2 with no change in Bcl-xL levels following over-expression of TIMP-1.

The classical function of all TIMPs is to inhibit matrix metalloproteinases [39]. However, the well-documented anti-apoptotic function of TIMP-1 has been demonstrated to be either MMP-independent or MMP-dependent [40]. Therefore we created a mutant form of TIMP-1, T2G that lacks MMP-inhibitory function. Mutant T2G was as effective as TIMP-1 in inhibiting apoptosis, indicating that the antiapoptotic function of TIMP-1 was MMP-independent in our system.

Since overexpressing Bcl-2 resulted in increased expression of TIMP-1 [21] and several studies have shown that overexpressing TIMP-1 resulted in upregulation of Bcl-xL (see above), a coordinated signaling loop between TIMP-1 and the Bcl-2 family was suggested in the regulation of apoptosis commitment step [18]. Our study provides evidence for the existence of such a feedback loop. The signaling feedback loop that appears to be functional between TIMP-1 and Bcl-2 is indeed striking; therefore we sought to identify a direct interaction between TIMP-1 and Bcl-2, and have shown that immunoprecipitation of Bcl-2 coimmunoprecipitates TIMP-1. Abrogating Bcl-2 activity using ABT-737 during induction of apoptosis with staurosporine, resulted in significant reduction of TIMP-1 protein and mRNA levels. Furthermore, this was specific for TIMP-1 as TIMP-2 levels were not altered, again reinforcing the fact that TIMP-1 and Bcl-2 are interdependent. It is not clear yet whether this is due to direct physical interaction between these two proteins or is mediated via other signaling proteins. TIMP-1 is known to translocate to the nucleus [41, 42] and Bcl-2 has also been found in the nucleus [43, 44], hence it is possible that they are both involved in transcriptional regulation. However, additional studies would be required to confirm such a role for these two proteins in the nucleus.

In most studies, signaling pathways leading to the antiapoptotic role of TIMP-1 have shown the involvement of FAK/PI-3/Akt pathway leading to phosphorylation of BAD at Ser-136. Phosphorylation of BAD at either Ser-112, Ser-136 or Ser-155 results in inactivation of this pro-apoptotic function. BAD phosphorylation at Ser-112 can occur through the RAS-RAF-MEK-ERK pathway [45], via phosphorylation of p90RSK, or by direct activation by RAF-1 [46]. It has also been shown that RSK can be activated by PDK1 without affecting Akt [47]. Activated p90RSK then phosphorylates BAD at Ser-112. Interestingly, in our lung carcinoma model, we have shown that BAD is phosphorylated at Ser-112 and not at Ser-136, thereby confirming our findings that TIMP-1 activated cell survival does not involve Akt activation.

H2009 cells carry a constitutively active KRAS [48]. Also, TIMP-1 is known to activate RAS and RAF-1 [49]. Thus it appears reasonable that this could then lead to downstream signaling through p90RSK. We found p90RSK to be phosphorylated at Ser-380, a site of autophosphorylation by its activated C-terminal kinase domain. We saw no phosphorylation at the Thr-573 site that gets activated directly by ERK. However, ERK involvement is still thought to occur indirectly [50]. Although we could not identify ERK activation in our study (Fig 4), this observation is not without precedent. It has previously been demonstrated that even if the MAPK pathway is active, p-ERK levels may not vary, as the levels of DUSP phosphatases are simultaneously elevated with increased MEK/ERK output, thus making any change difficult to detect [51]. Alternatively, oncogenic RAS cells may use effectors other than MAPK for cell survival [52]. Indeed, we were able to demonstrate activation of p90RSK along with phosphorylation of BAD. This cascade resulted in concomitant decrease in the levels of Bax, a pro-apoptotic protein that forms oligomers leading to the permeabilization of the mitochondrial outer membrane, activation of caspase 3 and finally a reduction in PARP cleavage. Furthermore, staurosporine treatment led to restoration of Bax expression.

TIMP-1 is a well-documented antiapoptotic marker as well as a prognostic marker in many cancers [5] particularly breast cancer [12]. TIMP-1 has also been shown to be of prognostic value in NSCL cancer [16]. High serum and tissue levels of TIMP-1 have been well correlated with shorter disease-free patient survival. TIMP-1 may also have predictive value as high TIMP-1 levels also correlate with decreased sensitivity to chemotherapeutic agents, thus indicating that TIMP-1 protects against apoptosis induced by chemotherapy [17]. Also it has been shown that TIMP-1 deficiency increases sensitivity to chemotherapy induced apoptosis in fibrosarcoma cells [53]. Additionally, in studies of liver fibrosis, a correlation has been seen between the levels of TIMP-1 and Bcl-2 [30, 32].

These studies necessitate a thorough dissection of the role of TIMP-1 in apoptosis. Although TIMP-1 has emerged as an important prognostic marker in clinical findings and a well-documented inhibitor of apoptosis in research studies, it is still unclear as to how TIMP-1 is upregulated in cancer. Our data provides evidence that TIMP-1 and Bcl-2 expression levels are interdependent and that these two proteins interact in vivo. This interaction may play a role in controlling the coordinated signaling loop. Additional studies are necessary to elucidate these interactions and could make TIMP-1 a viable and better therapeutic target.
